# Brain Injury Patterns and Short-TermOutcomes in Late Preterm Infants Treated with Hypothermia for Hypoxic Ischemic Encephalopathy

**DOI:** 10.3390/children12081012

**Published:** 2025-07-31

**Authors:** Aslihan Kose Cetinkaya, Fatma Nur Sari, Avni Merter Keceli, Mustafa Senol Akin, Seyma Butun Turk, Omer Ertekin, Evrim Alyamac Dizdar

**Affiliations:** 1 Department of Neonatology, Ankara Bilkent City Hospital, 06800 Ankara, Türkiye; 2 Department of Neonatology, Ankara Bilkent City Hospital, University of Health Sciences, 06800 Ankara, Türkiye; 3 Department of Pediatric Radiology, Ankara Bilkent City Hospital, 06800 Ankara, Türkiye

**Keywords:** hypoxic–ischemic encephalopathy, preterm, therapeutic hypothermia

## Abstract

**Background:** Hypoxic–ischemic encephalopathy (HIE) is a leading cause of severe neurological impairments in childhood. Therapeutic hypothermia (TH) is both safe and effective in neonates born at ≥36 weeks gestation with moderate to severe HIE. We aimed to evaluate short-term outcomes—including brain injury detected on magnetic resonance imaging (MRI)—in infants born at 34–35 weeks of gestation drawing on our clinical experience with neonates under 36 weeks of gestational age (GA). **Methods:** In this retrospective cohort study, 20 preterm infants with a GA of 34 to 35 weeks and a matched cohort of 80 infants with a GA of ≥36 weeks who were diagnosed with moderate to severe HIE and underwent TH were included. Infants were matched in a 1:4 ratio based on the worst base deficit in blood gas and sex. Maternal and neonatal characteristics, brain MRI findings and short term outcomes were compared. **Results:** Infants with a GA of 34–35 weeks had a lower birth weight and a higher rate of caesarean delivery (both *p* < 0.001). Apgar scores, sex, intubation rate in delivery room, blood gas pH, base deficit and lactate were comparable between the groups. Compared to infants born at ≥36 weeks of GA, preterm neonates were more likely to receive inotropes, had a longer time to achieve full enteral feeding, and experienced a longer hospital stay. The mortality rate was 10% in the 34–35 weeks GA group. Neuroimaging revealed injury in 66.7% of infants born at 34–35 weeks of gestation and in 58.8% of those born at ≥36 weeks (*p* = 0.56). Injury was observed across multiple brain regions, with white matter being the most frequently affected in the 34–35 weeks GA group. Thalamic and cerebellar abnormal signal intensity or diffusion restriction, punctate white matter lesions, and diffusion restriction in the corpus callosum and optic radiations were more frequently detected in infants born at 34–35 weeks of gestation. **Conclusions:** Our study contributes to the growing body of literature suggesting that TH may be feasible and tolerated in late preterm infants. Larger randomized controlled trials focused on this vulnerable population are necessary to establish clear guidelines regarding the safety and efficacy of TH in late preterm infants.

## 1. Introduction

Hypoxic–ischemic encephalopathy (HIE) is estimated to affect approximately 1.5 per 1000 live births in developed countries [1]. Its incidence ranges from 1 to 8 per 1000 live births and is reported to be higher in low- and middle-income countries [2]. Based on 2023 data from the Turkish Neonatal Society, the incidence of HIE among infants born at ≥36 weeks of gestation was reported as 2.13 per 1000 live births, representing 1.55% of neonatal intensive care unit (NICU) admissions [3]. Although the incidence of HIE is relatively low, it remains a significant cause of neonatal mortality and morbidity. Among survivors, around 25% experience severe neurological complications such as cerebral palsy [4], epilepsy, cognitive and intellectual impairments, as well as visual and hearing deficits [5].

Therapeutic hypothermia (TH) is the gold standard neuroprotective treatment for HIE, proven to reduce mortality and the risk of severe neurodevelopmental disabilities [6]. TH protects the brain by lowering cerebral metabolic demands, thereby minimizing secondary energy failure and neuronal death. It also helps mitigate hypoxic–ischemic injury by attenuating apoptosis, preserving mitochondrial function, and reducing oxidative stress and inflammation [7].

Previous studies have shown that TH is both safe and effective in neonates with ≥36 weeks’ gestation with moderate to severe HIE, by reducing the severity of neurological injury and improving survival [6,8,9,10,11]. The success of TH in neonates ≥ 36 weeks GA has led to growing interest in exploring its potential benefits for more premature infants. Although American Academy of Pediatrics (AAP) includes infants born at 35 weeks and beyond for TH, limited data is available regarding the clinical safety and effects of TH in infants born at less than 36 weeks of gestation [12]. According to the Vermont Oxford Network, the use of TH in infants born at less than 36 weeks of GA has increased over time, rising from 5.8% in 2012 to 8.2% in 2019 [13]. A recent multicenter randomized controlled trial evaluated the safety and efficacy of TH in infants born at 33 and 35 weeks of GA with moderate to severe HIE. The study aimed to determine whether initiating TH within six hours of birth would reduce the incidence of death or moderate to severe neurodevelopmental disability at 18 to 22 months’ corrected age. However, the findings did not demonstrate a significant reduction in either outcome [14]. In addition, this study is lacking to fill the gaps in the literature regarding MRI findings.

In this study, we aimed to evaluate short-term outcomes—including brain injury detected on magnetic resonance imaging (MRI) in infants born at 34–35 weeks of gestation with moderate to severe HIE who underwent TH.

## 2. Materials and Methods

In this retrospective cohort study, we included preterm infants with a GA of 34 to 35 weeks and a matched cohort of the infants with a GA of ≥36 weeks who were diagnosed with moderate to severe encephalopathy and underwent TH between October 2019 and December 2024 at a tertiary NICU in Ankara, Turkiye. Infants were matched in a 1:4 ratio based on sex and the worst base deficit in blood gas. The base deficit of the infants with a GA of 34 to 35 weeks was required to be within ±2 mmol/L of the base deficit of the corresponding infants with a GA of ≥36 weeks.This study was approved by the Institutional Review Board of Ankara Bilkent City Hospital. Parental informed consent was obtained for each infant before the initiation of TH.

Infants who met the criteria for TH, as outlined in the national guidelines for the management of HIE, were included in the study and received whole-body cooling.

Eligibility criteria:

(1) Infants born at ≥36 weeks’ gestation and ≤6 h of age;
(2) A pH value of ≤7.00 or a base deficit of ≤−16 mmol/L in cord blood gas or in a blood sample obtained within the first hour after birth;
(3) An Apgar score of ≤5 at 10 min or the need for prolonged resuscitation;
(4) An abnormal neurological examination consistent with moderate to severe encephalopathy.

If the pH was between 7.01 and 7.15, the base deficit was between −12 and −15.9 mmol/L, or if a blood gas sample was unavailable, additional criteria were required: evidence of an acute perinatal event (e.g., late or variable decelerations, cord prolapse) and either a 10 min Apgar score of ≤5 or the need for assisted ventilation initiated at birth and continued for at least 10 min.

In our NICU infants between 34^0/7^ and 35^6/7^ weeks of GA are offered TH as an individualized treatment, provided that all requirements are met.

Exclusion criteria: Infants under the GA of 34 weeks, weighing less than 2000 g, infants with severe congenital anomalies and severe intracranial hemorrhage.

We recorded the data for each infant including maternal and neonatal characteristics such as gestational age, birthweight, sex, mode of delivery, need for intubation in the delivery room, Apgar scores at 1, 5 and 10 min, umbilical cord blood gases and/or blood gases within the first hour of life, clinical findings and brain MRI results. Short term outcomes during hospitalization like hypoglycemia (glucose < 40 mg/dL), hyperglycemia (glucose ≥ 150 mg/dL), clinical and/or aEEG-confirmed seizure, hypotension [15], thrombocytopenia (platelet count < 150.000 × 10^9^/L), coagulopathy (prothrombin time > 16 s, international normalized ratio > 1.4, APTT > 79 s for 34–36 weeks of gestation and APTT > 55 s for ≥37 weeks of gestation), culture-proven late-onset sepsis (LOS) [16], acute renal injury [17], brain injury, survival to discharge were compared between the groups.

Neonates underwent whole-body TH according to national guideline, using servo control mode to maintain a rectal temperature of 33.5 ± 0.5 °C for 72 h. Following the 72 h TH period, rewarming was initiated, with core temperature increased at a controlled rate of 0.5 °C per hour. Infants underwent amplitude generated electroencephalography (aEEG) monitoring throughout both the cooling and rewarming phases.

To assess the presence and pattern of brain injury, MRI scans were performed after completion of TH and prior to hospital discharge, as part of routine clinical care. MRI studies were evaluated by a pediatric radiologist blinded to the infant’s clinical course using the qualitative scoring system described by Weeke et al. [18].

MRI scans were evaluated for injury in the following areas: (1) deep grey matter (thalamus, basal ganglia, posterior limb of the internal capsule, brainstem, perirolandic cortex, and hippocampus (2) cerebral white matter/cortex (cortex, cerebral white matter, optic radiations, corpus callosum, punctate white matter lesions, and parenchymal hemorrhage), and (3) cerebellum (cerebellum and cerebellar hemorrhage). The additional score describes the presence of intraventricular or subdural hemorrhage and sinovenous thrombosis. Each region was independently assessed for injury, and total score was generated by adding up the 4 regional sub-scores (grey matter + white matter +cerebellum + additional).

### Statistical Analysis

Statistical analysis was conducted using SPSS software for Windows (version 17.0; IBM, Chicago, IL, USA). The distribution of continuous variables was assessed using the Kolmogorov–Smirnov or Shapiro–Wilk tests. Continuous variables were compared using the Mann–Whitney U test and are presented as medians with interquartile ranges (IQR, 25th–75th percentiles). Categorical variables were analyzed using the chi-square test, with Fisher’s exact test applied when appropriate. Results for categorical variables are expressed as numbers and percentages. A two-sided *p*-value of <0.05 was considered statistically significant.

## 3. Results

The study cohort included 20 preterm infants with a GA of 34 to 35 weeks and a matched cohort of 80 infants with a GA of ≥36 weeks who were diagnosed with moderate to severe HIE and underwent TH.

Demographic characteristics are shown in Table 1. Two of the infants in the study were born at 34 weeks of gestation. Infants with a GA of 34–35 weeks had a lower birth weight and a higher rate of caesarean delivery (both *p* < 0.001). Delivery complications were comparable between the two groups, except that fetal decelerations and/or bradycardia were more frequently observed in the 34–35 weeks GA group (*p* = 0.045).

Table 2 presents the short term outcomes of the groups. Infants born at 34–35 weeks of gestation were more likely to receive inotropic support, had a longer to time achieve full enteral feeding, and experienced a longer hospital stay compared to those born at ≥36 weeks.

The overall mortality rate was 3%, with a significantly higher rate observed in the 34–35 weeks GA group (*p* = 0.040).

Median (IQR) age at MRI scan was 5 (4–8) days in 34–35 weeks GA group and 6 (5–7) days in ≥36 weeks GA group (*p* = 0.82). Neuroimaging revealed injury in 66.7% of infants born at 34–35 weeks of GA and in 58.8% of those born at ≥36 weeks of GA (*p* = 0.56). Weeke total scores, as well as injuries to the grey matter, white matter, cerebellum, intraventricular hemorrhage (IVH), subdural hemorrhage (SDH), and cerebral venous sinus thrombosis (CVST) on MRI, were similar between the groups. However, neuroimaging findings revealed significantly more frequent abnormalities in the 34–35 weeks GA group, including thalamic and cerebellar abnormal signal intensity or diffusion restriction, punctate white matter lesions (PWML), and diffusion restriction in the corpus callosum and optic radiations (*p* = 0.031, 0.029, 0.002, 0.033 and 0.004, respectively). In the preterm group, injuries were observed across all brain regions, with white matter being the most commonly affected. The patterns of brain injury observed on MRI are detailed in Table 3.

## 4. Discussion

In this study, preterm infants born at 34–35 weeks of GA with moderate to severe HIE were more likely to require inotropic support, had a longer time to achieve full enteral feeding, and experienced prolonged hospital stay. Mortality was not found to be high in our whole cohort; however, it was notably higher in infants with lower GA compared to more mature ones. Neuroimaging findings revealed abnormalities more frequently in the 34–35 weeks GA group, including thalamic and cerebellar abnormal signal intensity or diffusion restriction, PWML, diffusion restriction in the corpus callosum and optic radiations.

TH offers significant advantages in enhancing survival rates and neurodevelopmental outcomes, which outweigh the potential risks associated with short-term complications. TH therapy in infants born before 36 weeks of GA with moderate to severe HIE has increased in recent years [12,19,20,21]. A recent multicenter randomized controlled trial evaluated the safety and efficacy of TH in infants born at 33–35 weeks’ GA with moderate to severe HIE. The study aimed to determine whether initiating TH within six hours of birth could reduce the incidence of death or moderate to severe neurodevelopmental disability by 18 to 22 months’ corrected age. However, the results showed no significant reduction in either outcome. A key criticism of the trial is the lack of subgroup analysis for infants born at 34 and 35 weeks, as well as the absence of MRI data [14].

Current evidence supporting the use of TH in preterm infants is limited to retrospective studies, case series, and registry data. Moran et al. conducted a single-centre retrospective study involving 42 preterm infants born at <36 weeks’ GA (range: 32–35 weeks) with moderate-to-severe HIE who received TH. While the incidence of TH-related morbidities was comparable with term infants, inotrope requirement was higher preterm infants [20] similar to our study. Although the rate was lower than that reported by Moran et al., it was due to the inclusion of more immature infants in their cohort. Hypotension in preterm infants may result from hemodynamic instability, a reflection of their heightened vulnerability due to immature cardiovascular physiology. Thus, the observed hemodynamic instability may be attributable more to prematurity itself than to the effects of TH. In a study by Herrera et al., 30 infants with a median GA of 35 weeks (range: 33–35 weeks) who underwent TH over an eight-year period were evaluated. Coagulopathy requiring treatment emerged as the most frequent complication, observed in 50% of cases significantly higher than the rates reported in the three hypothermia trials (18–41%) [9,10,22]. Hypotension necessitating inotropic support was the second most common complication observed during TH therapy [12]. In another study, Kim et al. reported comparable short-term outcomes in preterm infants who underwent TH, consistent with our findings [21].

Mortality rates among preterm infants treated with TH vary considerably across the studies referenced above, likely reflecting differences in GA, severity of encephalopathy, study design, and the period during which data were collected. Moran et al. reported a mortality rate of 24% in the preterm cohort, which was comparable to 19% in the term group. The authors attributed the relatively high mortality to the early years of TH implementation, as their study dates back to 2005 [20]. In a national population-based study, Okulu et al. identified 46 preterm infants born at 34–35 weeks’ gestation with HIE among 1011 cases. TH was administered to 67.4% of these infants, most of whom presented with moderate to severe HIE. The mortality rate was significantly higher among infants born at 34–35 weeks (21.7%) compared to term infants (6%) [3]. Rao et al. reported an overall mortality of 12.9%, with all deaths occurring in the preterm cohort—half of whom were diagnosed with severe HIE [19]. Also, Lademann et al. reported a mortality rate of 13% among preterm infants and 19% among term infants who received TH, while the overall mortality among all asphyxiated infants in their cohort was 8% [23]. These figures are lower than the 30% mortality rate documented in a Cochrane meta-analysis of eleven randomized controlled trials, which included 1468 infants and 436 deaths [6]. Conversely, Kim et al. reported no deaths in the 34–35 weeks GA group. In their entire cohort of 60 infants, only two deaths occurred, resulting in a lower overall mortality rate compared to other published studies [21]. Our study similarly demonstrated a relatively low mortality rate. Mortality in our whole cohort was 3%, increasing to 10% among more premature infants, yet remaining notably lower than previously reported rates. Several factors may explain this favourable outcome. First important contributor is the implementation of national guidelines developed by the Turkish Neonatal Society, which offer structured protocols for newborn care and resuscitation. Additionally, most of the infants in our study were inborn, suggesting a likely benefit from timely and standardized perinatal care. Optimal antepartum and intrapartum management, broader access to TH, and proper neonatal intensive care have likely contributed to the observed reduction in mortality. It is also considered that the low number of infants born at 34 weeks of gestation might have contributed to the lower mortality rate.

During the neonatal period, MRI is widely used to assess brain injury in cases of HIE. Despite its importance, many MRI scoring systems do not incorporate diffusion-weighted imaging (DWI), even though DWI has proven to be the most sensitive sequence for detecting brain injury within the first week following a hypoxic–ischemic insult. Previous studies have emphasized that the pattern of injury differs between preterm and term infants with HIE. In preterm infants, the injury predominantly affects the cerebral white matter, often presenting as periventricular leukomalacia (PVL), which disrupts oligodendrocyte maturation and impairs subsequent myelination [24,25]. In contrast, term infants are more prone to neuronal injury involving the cerebral cortex, basal ganglia, and thalamus, with selective neuronal necrosis [26,27]. In our study, brain injury was assessed using the novel scoring system proposed by Weeke et al. (18). Most traditional MRI scoring systems are based solely on T1- and T2-weighted sequences, typically acquired during the second week of life, as abnormalities may not be evident earlier. In contrast, DWI is highly effective in the first week of life—a crucial period for clinical decision making and potential initiation of additional neuroprotective therapies. DWI allows for early detection of cytotoxic edema, often identifying lesions several days before they become visible on T1 or T2 sequences [28,29].

Kim et al. found no significant differences in total Weeke scores or the prevalence of grey matter, white matter, or cerebellar injury between infants born at 34–35 weeks and those ≥36 weeks of gestation, a finding consistent with our results [21]. Conversely, we observed that 34–35 weeks GA group had abnormalities on MRI scans, including thalamic and cerebellar signal abnormalities or diffusion restriction, PWML, and restricted diffusion in the corpus callosum and optic radiations. PWML has been reported in approximately 10–20% of neonates with neonatal encephalopathy or HIE, predominantly in near-term rather than full-term infants [30,31]. This is relatively common pattern in preterm neonates and those presenting with milder forms of encephalopathy [32]. Hayakawa et al. observed diffusion restriction in the optic radiation in 29% of term neonates with HIE at a median age of 3.5 days. This finding is not uncommon in term infants with HIE, with DWI typically revealing changes during the first week, followed by high-intensity T1 signal abnormalities during the second week [33]. Kale et al. found restricted diffusion in the corpus callosum in 16 out of 40 patients presented with HIE and they proposed that this finding likely represents acute cytotoxic edema and may reflect a generalized response to hypoxic–ischemic injury [34]. In addition, Rao et al. reported brain injury in 80.6% of preterm and 59.4% of term infants, with white matter injury being significantly more frequent in the preterm group (66.7% vs. 25.0%, *p* = 0.001) [19]. In our preterm group, injury was observed across multiple brain regions, with white matter being the most frequently affected. This pattern likely reflects both the lower gestational age and the heightened vulnerability of the white matter and cerebellum at this stage of development. Binet et al. evaluated brain lesions according to GA in newborns with neonatal encephalopathy and found that white matter lesions were more prevalent in preterm infants compared to those born at term similar to our study [35]. Also, Logitharajah et al. analyzed MRI findings in 55 preterm infants (26–36 weeks gestation). They found white matter injury in 89%, basal ganglia and thalamic injury in 72%, cortical injury in 47%, and brainstem injury in 43% of cases. These findings further support the notion that white matter in preterm infants is particularly susceptible to hypoxic–ischemic injury [36].

The major strength of our study is that it provides data on infants who underwent TH due to moderate to severe HIE, a population for which limited information is available. The main limitations of our study are the small sample size, the limited number of infants born at 34 weeks of gestation, its retrospective design, and the lack of data on long-term outcomes. Additionally, demographic features in the late preterm infants suggest that the etiology of HIE may differ between the two groups. Since there is a risk of pseudonormalization on diffusion imaging after 7 days of life, the different postnatal days on which MRI was performed should be taken into account when interpreting the results. The days to initiation of enteral feeds was also not recorded, so it is unclear whether the time of FEF is a reflection of a physiologic difference between the groups or a hesitance on the part of the clinicians to start enteral feeds for the late preterm group.

## 5. Conclusions

Preterm infants born at 34–35 weeks of GA who underwent TH did not show an increased risk of major morbidities compared to infants born at ≥36 weeks, and TH was well tolerated in this group. However, our findings suggest that this population may be more susceptible to white matter injury following HIE. Notably, mortality in the 34–35 weeks GA group was higher compared to the overall cohort, emphasizing the vulnerability of this population. Given these findings, further studies are essential to provide more robust evidence before TH can be confidently recommended for these higher-risk infants.

## Figures and Tables

**Table 1:** Demographic characteristics of the groups.

	34–35 Weeks GA n = 20	35 Weeks GA n = 18	≥36 Weeks GA n = 80	*p*-Value
GA (weeks) *	35 (35–35)	35 (35–35)	39 (38–40)	<0.001
Birthweight (g) *	2510 (2275–3035)	2640 (2295–3102)	3240 (2965–3490)	<0.001
Male, n (%)	8 (40)	7 (39)	30 (37.5)	0.83
1 min. Apgar score *	4.5 (2–6)	4 (2–6)	5 (3–6)	0.32
5 min. Apgar score *	7 (6–8)	6.5 (4–8)	7 (4.5–8)	0.54
10 min. Apgar score *	7 (5–8)	7 (5–8)	7 (6–8)	0.60
Intubation in DR	7 (35)	7 (39)	19 (23.8)	0.30
Blood gas *				
pH	6.97 (6.79–6.98)	6.90 (6.70–7.00)	6.97 (6.90–7.04)	0.17
Base deficit (mmol/L)	−17.6 [(−24.5)–(−16.2)]	−17.1 [(−24.2)–(−15.7)]	−16.4 [(−20.8)–(−14.5)]	0.11
Lactate (mmol/L)	8.9 (5–12)	9.1 (5.5–12.1)	8.4 (5.6–11.2)	0.93
Grade of HIE				
Moderate	16 (80)	14 (77.8)	75 (93.8)	0.055
Severe	4 (20)	4 (22.2)	5 (6.2)	
Outborn, n (%)	3 (15)	3 (16.7)	24 (30)	0.17
Caesarean delivery, n (%)	19 (95)	17 (94.4)	36 (45)	<0.001
Pregnancy complications, n (%)				
Preeclampsia	1 (5)	1 (5.6)	1 (1.3)	0.28
Diabetes	0 (0)	0 (0)	3 (3.8)	0.37
Hypothyroidism	1 (5)	0 (0)	3 (3.8)	0.79
Preterm rupture of membranes	1 (5)	1 (5.6)	4 (5)	1.00
Delivery complications, n (%)				
Uterine rupture	0 (0)	0 (0)	1 (1.3)	0.61
Placental abruption	1 (5)	0 (0)	5 (6.3)	0.83
Cord prolapse	0 (0)	0 (0)	2 (2.5)	0.47
Fetal decelerations/bradycardia	5 (25)	5 (27.8)	7 (8.8)	0.045
Meconium presence	1 (5)	1 (5.6)	14 (17.5)	0.16

Data given as * median (IQR); DR: delivery room, GA; gestational age.

**Table 2:** Short-term outcomes of the groups.

	34–35 Weeks GA n = 20	35 Weeks GA n = 18	≥36 Weeks GA n = 80	*p*-Value
Hypoglycemia, n (%)	5 (25)	4 (22.2)	8 (10)	0.057
Hyperglycemia, n (%)	3 (15)	3 (16.6)	7 (9.2)	0.35
Abnormal coagulation, n (%)	11 (55)	11 (61)	32 (46.4)	0.26
Thrombocytopenia, n (%)	5 (25)	5 (27.8)	11 (13.8)	0.22
Hypotension/inotropic support, n (%)	8 (40)	8 (44.4)	8 (10)	0.001
RBC transfusion, n (%)	2 (10)	2 (11.1)	6 (7.5)	0.71
Platelet transfusion, n (%)	3 (15)	3 (16.7)	9 (11.3)	0.64
Fresh frozen plasma transfusion, n (%)	4 (20)	4 (22.2)	15 (18.8)	0.89
Cryoprecipitate transfusion, n (%)	0 (0)	0 (0)	2 (2.5)	0.47
Intubation during hospitalization, n (%)	13 (65)	12 (66.7)	28 (35)	0.015
Seizures, n (%)	10 (50)	9 (50)	25 (31.3)	0.11
Acute renal injury, n (%)	1 (5)	1 (5.6)	5 (6.3)	0.83
Culture proven LOS, n (%)	2 (10)	2 (11.1)	5 (6.3)	0.55
Time of FEF (days) *	8 (6–10)	7.5 (6–10)	5 (4–7)	0.004
Length of stay (days) *	16 (12–25)	16 (12–24)	10 (7–15)	0.002
Mortality, n (%)	2 (10)	2 (11.1)	1 (1.3)	0.040

Data given as * median (IQR); FEF: full enteral feeding, LOS: late-onset sepsis, RBC: Red blood cell.

**Table 3:** MRI patterns of brain injury of the groups.

	34–35 Weeks GA n = 17	≥36 Weeks GA n = 80	*p*-Value
Grey matter injury on MRI	4 (23.5%)	17 (21.3%)	0.836
Thalamus abnormal signal intensity or diffusion restriction	3 (17.6%)	3 (3.8%)	0.031
Basal ganglia abnormal signal intensity or diffusion restriction	3 (17.6%)	9 (11.3%)	0.467
PLIC myelination or diffusion restriction	3 (17.6%)	8 (10%)	0.367
Brainstem (peduncles) abnormal signal intensity or diffusion restriction	1 (5.9%)	1 (1.3%)	0.222
Perirolandic cortex diffusion restriction	2 (11.8%)	8 (10%)	0.828
Hippocampus diffusion restriction	1 (5.9%)	1 (1.3%)	0.222
White matter/cortex injury on MRI	8 (47.1%)	29 (36.3%)	0.405
Cortex abnormal signal intensity or diffusion restriction not being perirolandic cortex	0 (0)	1 (1.3%)	0.643
White matter increased signal intensity or diffusion restriction not being PWML	7 (41.2%)	18 (22.5%)	0.110
PWML	3 (17.6%)	1 (1.3%)	0.002
Hemorrhage not being PWML	2 (11.8%)	14 (17.5%)	0.563
Optic radiation diffusion restriction	4 (23.5%)	3 (3.8%)	0.004
Corpus callosum diffusion restriction	5 (29.4%)	8 (10%)	0.033
Cerebellum injury on MRI	2 (11.8%)	2 (2.5%)	0.081
Cerebellum abnormal signal intensity or diffusion restriction	1 (5.9%)	0 (0)	0.029
Cerebellar hemorrhage	1 (5.9%)	2 (2.5%)	0.464
IVH	1 (5.9%)	3 (3.8%)	0.688
SDH	5 (29.4%)	12 (15%)	0.156
CSVT	0 (0)	2 (2.5%)	0.510
Weeke total score	2 (0–4)	1 (0–2)	0.145

CSVT cerebral sinovenous thrombosis; IVH intraventricular hemorrhage; PLIC posterior limb of the internal capsule; PWML punctate white matter lesions; SDH subdural hemorrhage.

## Data Availability

The original contributions presented in the study are included in the article, further inquiries can be directed to the corresponding author.
